# Effects of Phytochemicals on Type 2 Diabetes via MicroRNAs

**DOI:** 10.1007/s13668-024-00549-5

**Published:** 2024-05-28

**Authors:** Dilek Sivri, Makbule Gezmen-Karadağ

**Affiliations:** 1 Department of Nutrition and Dietetic, Faculty of Health Science, Anadolu University, Eskişehir, Turkey; 2 Department of Nutrition and Dietetic, Faculty of Health Science, Gazi University, Ankara, Turkey

**Keywords:** Type 2 Diabetes, Insulin resistance, MicroRNA, Phytochemicals

## Abstract

**Purpose of Review:**

Type 2 diabetes, characterized by inadequate insulin secretion and resistance, is increasingly prevalent. To effectively manage type 2 diabetes, identifying new therapeutic targets is crucial. MicroRNAs, short noncoding RNA molecules, play a pivotal role in regulating β-cell function, insulin production, and resistance, and show promise as biomarkers for predicting type 2 diabetes onset. Phytochemicals, known for their antioxidant activities, may influence microRNA expression, potentially improving insulin sensitivity and mitigating associated complications. This review aims to explore the significance of microRNA in type 2 diabetes, their potential as biomarkers, and how certain phytochemicals may modulate microRNA expressions to reduce or prevent diabetes and its complications.

**Recent Findings:**

Current research suggests that microRNAs show promise as novel therapeutic biomarkers for diagnosing type 2 diabetes and monitoring diabetic complications. Additionally, phytochemicals may regulate microRNAs to control type 2 diabetes, presenting a potential therapeutic strategy.

**Summary:**

The multifactorial effects of phytochemicals on type 2 diabetes and its complications through microRNAs warrant further research to elucidate their mechanisms. Comprehensive clinical trials are needed to assess the safety and efficacy of phytochemicals and their combinations. Given their ability to modulate microRNAs expression, incorporating phytochemical-rich foods into the diet may be beneficial.

## Introduction

Type 2 diabetes is a chronic disease characterized by insufficient insulin release from pancreatic β cells or insulin resistance. As many as 425 million people were said to have had type 2 diabetes in 2017, and this number is expected to rise to 629 million by 2045 [1]. Genetic, epigenetic, environmental, and lifestyle factors influence the pathogenesis of type 2 diabetes. Epigenetics plays a crucial role in developing type 2 diabetes and insulin function. Toll-like receptors, nuclear factor kappa-B (NF-κB), and osteopontin are molecular components that undergo epigenetic regulation in the context of insulin resistance [2••].

An increasing pool of evidence points to the critical role of microRNA (miRNA) in metabolism and metabolic disorders, which has led to numerous studies focusing on the role of miRNAs in controlling energy metabolism in individuals with type 2 diabetes [2••, 3••]. Several studies have demonstrated the involvement of miRNAs in the onset and development of type 2 diabetes, highlighting their role in regulating cell function, insulin secretion, and insulin signaling pathways in target tissues. Alterations in miRNA expression profiles have been observed in type 2 diabetes, suggesting their potential as biomarkers for diagnosis and treatment [4••].

Phytochemicals, found in fruits, vegetables, whole grains, nuts, legumes and various plants, are bioactive compounds produced by plants for protection. Key phytochemicals include carotenoids, polyphenols, isoprenoids, phytosterols, saponins, dietary fibers, and certain polysaccharides. These compounds possess a range of beneficial properties, such as antioxidant, anti-inflammatory, anticarcinogenic, antimicrobial, antiallergic, antiaging, and antiviral activities. Additionally, they regulate gut microbiota, interact with hormonal receptors, enhance immunity, and modulate gene transcription [5•, 6].

Many drugs employed in the treatment of type 2 diabetes are associated with numerous side effects. Phytochemicals may provide a promising approach to alleviate and manage symptoms associated with type 2 diabetes. Their potential lies in mitigating oxidative stress, damage and injury, scavenging reactive oxygen species, and effectively modulating epigenetic mechanisms [2••, 3••].

This review aims to provide comprehensive information regarding the mechanisms of action of miRNAs in the pathogenesis, diagnosis, and treatment of type 2 diabetes. Furthermore, it will explore their potential use as biomarkers and the effectiveness of certain phytochemicals in reducing or preventing type 2 diabetes complications through the regulation of miRNAs expressions.

## The Importance of miRNAs in Type 2 Diabetes

Non-coding RNAs (ncRNAs), transcripts that are copied from DNA but do not translate into protein products, play a crucial role in physiological and pathological processes in various ailments, including cancer, diabetes, neurodegenerative diseases, immunodeficiency, and cardiovascular diseases. Dysregulation or defects in ncRNAs can contribute to the development of these diseases. Given their diverse functionality, ncRNAs have garnered significant interest as potential biomarkers and therapeutic tools in novel therapeutic approaches [7].

**Fig. 1:**
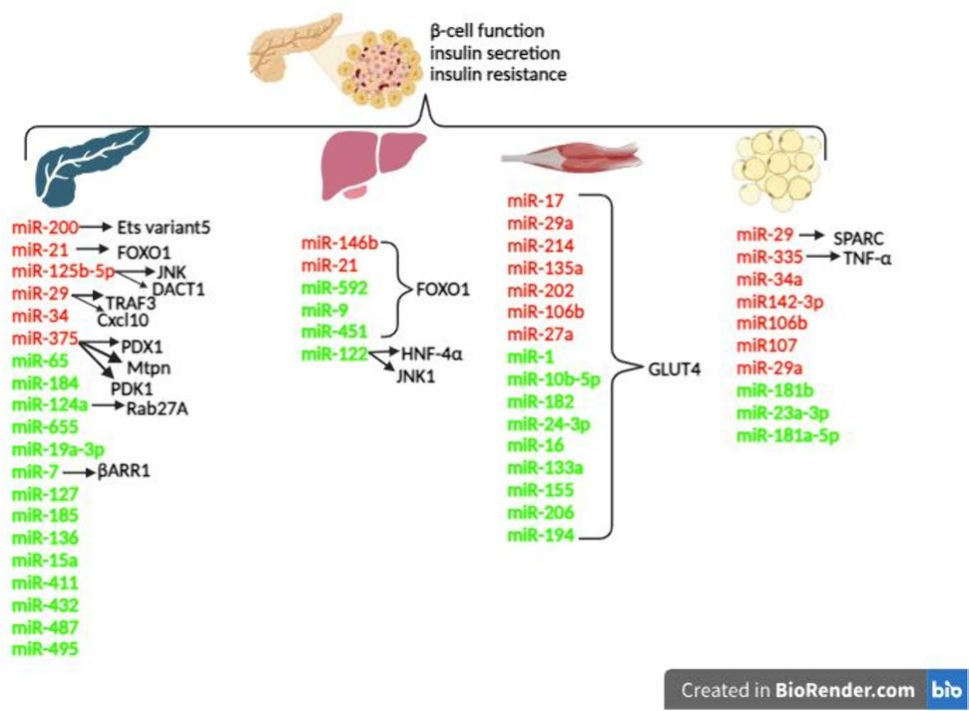
Specific miRNAs up/down regulated in the pancreas, liver, skeletal muscle, and adipose tissue play crucial roles in regulating β-cell function, insulin secretion, and insulin resistance through various pathways. Downregulated miRNAs are highlighted in green, upregulated miRNAs are highlighted in red. The roles of miRNAs are displayed in no particular order of importance. TRAF3: Tumor Necrosis Factor Receptor-Associated Factor 3, Cxcl10: C-X-C Motif Chemokine Ligand 10, JNK: c-Jun NH2-Terminal Kinase, DACT1: Disheveled Antagonist Dapper1, PDK1: Phosphoinositide-Dependent Protein Kinase 1, Rab27a: Ras-related Protein Rab-27a, βARR1: β-arrestin 1, Mtpn: Myotrophin, HNF-4α: Hepatocyte Nuclear Factor 4 Alpha, FOXO1: Forkhead Family of Transcription Factor, GLUT4: Glucose Transporter Member 4, SPARC: Secreted Protein Acidic and Rich in Cysteine, TNF: Tumor Necrosis Factor, PDX1: Pancreatic and Duodenal Homeobox-1

MiRNAs, a subset of ncRNAs, play crucial roles in numerous biological processes, with a single miRNA capable of targeting hundreds of genes. Dysregulation of miRNAs, which govern cellular differentiation, proliferation, apoptosis, and homeostasis, is implicated in various pathophysiological conditions. This suggests that miRNAs could represent a novel target for preventive or therapeutic interventions [8, 9]. In recent years, the rapid progression of miRNA research has revealed their importance in metabolic control. Some miRNAs have been identified that mediate adipocyte differentiation and function and control β cell function and insulin secretion in the pancreas, liver, skeletal muscle, and adipose tissue [9, 10••] (Fig. 1).

## Role of miRNAs in β-Cell Function, Insulin Production and Secretion

miRNA molecules play crucial roles in the functions of β-cells. Several pancreatic miRNAs, including miR-125a-5p, miR-21, miR-34, miR-200, and miR-375 are upregulated while miR-7, miR-184, miR-124a, miR-655, miR-19a-3p, miR-656, miR-127, miR-185, miR-136, miR-543, miR-15a, miR-411, miR-432, miR-487, and miR-495 are significantly downregulated in pancreatic islet function [10••] (Fig. 1).

miR-7 regulate glucagon-like peptide-1 (GLP-1), is an incretin hormone that stimulates insulin secretion in response to elevated blood glucose levels, signaling. miR-7 regulates insulin release by targeting β-arrestin 1 (βARR1), a protein involved in the desensitization of GLP-1 receptors. By inhibiting the expression of βARR1, miR-7 promotes GLP-1 signaling and enhances insulin secretion from pancreatic β-cells in response to glucose stimulation. Understanding the regulatory role of miR-7 in the GLP-1 pathway provides insights into potential therapeutic strategies for the treatment of diabetes [11].

miR-375, predominantly localized in the pancreas, serves multiple functions in the islet β-cell, including development, proliferation, and secretion roles. Most studies indicate upregulation of miR-375 in islets of type 2 diabetes, healthy human donors, and other murine models of diabetes [4••, 12]. It is considered a typical miRNA that needs to be expressed at optimal levels in the cell [13].

Several miRNAs regulate insulin production, with miR-375 notably inhibiting 3'-phosphoinositide-dependent protein kinase 1 (PDK1). The reduced levels of PDK1 contribute to the down-regulation of insulin gene expression in response to glucose stimulation [10••]. miR-375, crucial in regulating insulin secretion, is the most highly expressed miRNA in pancreatic islets. The target of miR-375 is myotrophin (Mtpn), which contributes to cytoskeletal remodeling by depolymerizing actin filaments and mediates exocytosis by facilitating the fusion of insulin vesicles to the membranes of β-cells. Additionally, Mtpn has been demonstrated to upregulate NF-κB, thereby inducing the expression of proteins responsible for directing insulin vesicles to the membrane [10••]. Another mechanism involves miR-375 limiting the stimulation of pancreatic and duodenal homeobox-1 (PDX1) activation by glucose through the PI-3 kinase pathway by suppressing PI 3-kinase signaling [12]. Changes in miR-375 levels in the blood have been reported five years before the onset of prediabetes and type 2 diabetes in studies on human populations [14].

The miRNA-200 family, consisting of miR-200a, miR-200b, miR-200c, miR-141, and miR-429, is expressed in pancreatic islet cells and regulates β-cell survival and insulin secretion [4••]. In type 2 diabetes islets, miR-200c expression increases, leading to reduced glucose-stimulated insulin secretion in EndoC-βH1 cells compared with non-diabetic donors. miR-200c targets Ets variant5 in human islets, suppressing insulin exocytosis and contributing to reduced insulin secretion, a vital phenotype observed in patients with type 2 diabetes [15•].

Mounting evidence suggests that the miR-29 family, comprising miR-29a, miR-29b-1, miR-29b-2, and miR-29c, may serve as critical markers of beta-cell function and could potentially play a significant role in the pathogenesis of diabetes and prediabetes [4••, 16]. The expression of miR-29 family members is upregulated in type 2 diabetes. A study demonstrates that miR-29 targets tumor necrosis factor (TNF) receptor-associated factor 3 to enhance Cxcl10 release from β cells, promoting islet inflammation. This process involves the recruitment of monocytes and macrophages through the TNF receptor-associated factor 3 (TRAF3) and C-X-C motif chemokine ligand 10 (Cxcl10) pathway. Therapeutic strategies aimed at inhibiting the effects miR-29 have been suggested as a potential approach to alleviate inflammation and diabetes [17].

In mice with type 2 diabetes, there is a diminished expression of miR-125b-5p but enhanced expressions of disheveled antagonist dapper1(DACT1), c-Jun NH2-terminal kinase (JNK), and c-Jun. miR-125b-5p inhibits DACT1 expression and the activation of the JNK signaling pathway. Upregulation of miR-125b-5p promotes insulin sensitivity and enhances pancreatic β-cell function by inhibiting the JNK signaling pathway through negative mediation of DACT1 [18].

## Role of miRNAs in Insulin Resistance

Insulin resistance refers to the impaired responsiveness of tissues, such as muscles, fat, and liver, to insulin at its average concentration. Dysregulation of miRNA contributes to insulin resistance in various target tissues, including the liver, skeletal muscle, and adipose tissue [19••] (Fig. 1).

The liver-specific miRNA, miR-122, is down-regulated by overexpression of JNK1. Inactivation of hepatocyte nuclear factor 4 alpha (HNF-4α) phosphorylation contributes to hepatic insulin resistance. Administration of JNK1 inhibitors can increase miR-122 expression, ameliorating insulin resistance [19••].

In hepatocytes, the forkhead family of transcription factor (FOXO1) induces the transcription of gluconeogenic enzymes such as glucose-6-phosphatase (G6Pase) and phosphoenolpyruvate carboxykinase (PEPCK), thereby promoting hepatic glucose production [4••]. FOXO1 is upregulated in the livers of obese individuals. In the livers of mice with high-fat diet-induced obesity, hepatic miR-592 was reduced, while FOXO1 mRNA was increased [20]. Downregulation of miR-9 leads to upregulation of FOXO1 and activation of hepatic gluconeogenesis [21]. Conversely, upregulation of miR-146b [22] and miR-21 [23] decreases FOXO1 expression, suppressing hepatic glycogenesis and response to insulin resistance induction. Furthermore, overexpression of miR-451 significantly enhances hepatic FOXO1 phosphorylation [24].

miRNAs have been implicated in insulin resistance in skeletal muscle and are dysregulated in both in vivo and in vitro studies. Some miRNAs, such as miR-17, miR-29a, miR-214, miR-135a, miR-202, miR-106b, and miR-27a, show increased expression, while others, such as miR-1, miR-10b-5p, miR-182, miR-24-3p, miR-16, miR-133a, miR-155, miR-206 and miR-194, exhibit decreased expression in skeletal muscle insulin resistance [4••, 19••, 25].

Elevated expression of miR-17 was observed in skeletal tissues of rats with type 2 diabetes. Downregulation of miR-17 resulted in the amelioration of glucose metabolism, accompanied by an elevation of glucose transporter member 4 (GLUT4) protein level [26]. GLUT4 is crucial in mediating insulin-induced glucose uptake by muscle and fatty tissues. Reduced GLUT4 expression/translocation is observed in prediabetes and diabetes [25]. The miRNAs miR-21a-5p, miR-27a, miR-29a-3p, miR-29c-3p, miR-30d, miR-93-5p, miR-106b, miR-133a-3p, miR-133b-3p, miR-222-3p, and miR-223-3p have been reported to directly and/or indirectly regulate GLUT4 expression [25, 27].

Given the critical role of miRNAs in regulating gene expression within cells, dysregulation of miRNAs can significantly alter adipocyte functions, contributing to obesity and insulin resistance [4••]. The miR-29 family members were found to reduce the secreted protein acidic and rich in secreted protein acidic and cysteine (SPARC) protein level, glucose uptake, and GLUT4 level in 3T3-L1 adipocytes [28]. The expression of miR-181b [29], miR-23a-3p, and miR-181a-5p [30] was found to be reduced in the adipose tissue of obese mice. These miRNAs are essential in regulating glucose homeostasis and insulin sensitivity in adipose tissue [29, 30]. TNF-α up-regulates miR-335 in adipocytes, leading to the down-regulation of genes involved in insulin signaling and lipid metabolism. Consequently, miR-335 serves as a link between inflammation and impaired metabolism in adipose tissue [31].

In a case-control study involving 574 patients with type 2 diabetes and 596 controls, the relationship between the miR-146a rs2910164 polymorphism and the risk of type 2 diabetes was evaluated, and the findings suggested that there was no statistical difference in genotype frequencies between the type 2 diabetes and control groups. In the meta-analysis part of the same study, it was emphasized that there was an increase in miR-34a/142-3p/106b/107 levels with an increase in miR-29a levels in fatty tissues of type 2 diabetes patients and obese/insulin resistance animal models [32•]. Furthermore, three meta-analyses evaluating miRNAs as potential biomarkers in type 2 diabetes suggested that miR-29a is a common candidate [33, 34, 35••]. This data supports the potential use of miR-29a in clinical trials for type 2 diabetes management.

## Circulating miRNAs in Type 2 Diabetes

**Fig. 2:**
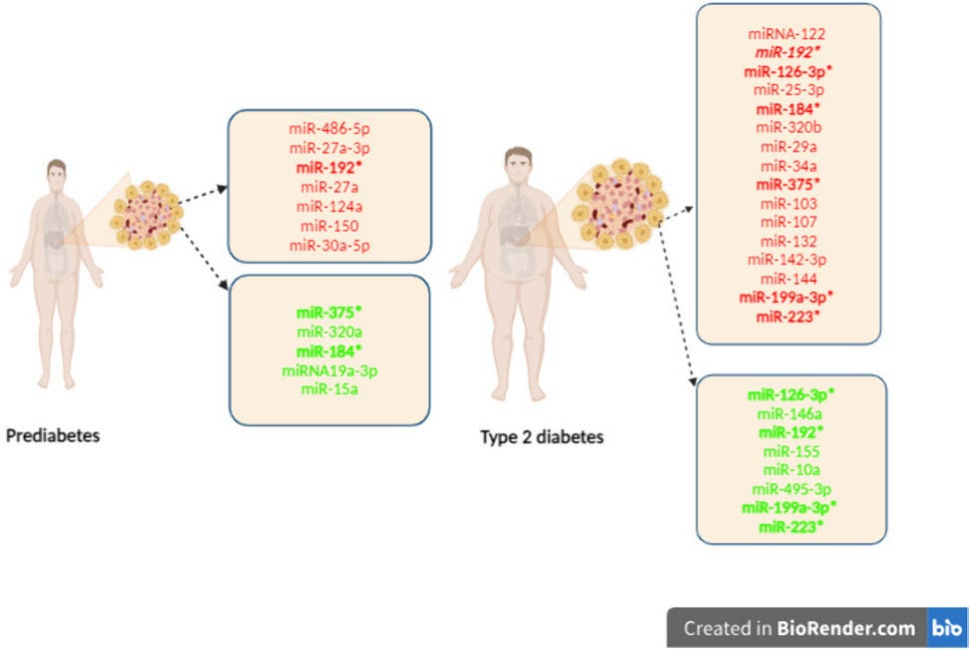
Schematic representation of miRNAs with altered expression in in prediabetes and type 2 diabetes in studies. Downregulated miRNAs are highlighted in green, upregulated miRNAs are highlighted in red. Asterisks (*) denote miRNAs with conflicting results. miR-192 expression increases in prediabetes but varies in type 2 diabetes. miR-375 and miR-184 decrease in prediabetes but increase in type 2 diabetes. Expression of miR-126-3p, miR-199a-3p, and miR-223 is variable in type 2 diabetes

The functions of many miRNAs on target tissues and metabolism have yet to be fully elucidated [9]. However, miRNAs can be detected in various body fluids, including plasma, serum, saliva, breast milk, urine, and seminal fluid. Altered miRNA profiles in circulation have been reported to be associated with metabolic diseases, including type 2 diabetes [9, 36]. Recent studies have revealed that circulating ncRNAs play a significant role in regulating glucose metabolism, insulin resistance, monitoring inflammation, and oxidative stress. These findings suggest that ncRNAs hold promise as novel therapeutic biomarkers for diagnosing type 2 diabetes and monitoring diabetic complications. The expression of specific miRNAs is altered in prediabetes and type 2 diabetes [36, 37, 38•] (Fig. 2).

## Circulating miRNAs as Risk Factors and Predictors for Type 2 Diabetes

In recent years, several studies have identified dysregulated circulating miRNAs in the serum/plasma of individuals with obesity, prediabetes, and insulin resistance [14, 26, 38•, 39]. In a cohort study on healthy adults, it was found that miR-320a targets the mRNA of Peroxisome Proliferator-Activated Receptor-Gamma (PPAR-ɣ) while FOXO1 and FOXO3 are targeted by miR-486-5p. miR-375 decreased in insulin resistant as opposed to insulin sensitive individuals. These miRNAs contribute to the enhancement of insulin resistance [39]. Studies have reported that miR-27a-3p levels increase in the presence of obesity [38•, 40]. It has also been reported that miR-27a-3p levels are high in pre-diabetic obese individuals and may serve as a biomarker in the onset of diabetes [38•].

Secretion of miRNAs into the blood can reveal low-grade alterations in organs. For example, miRNAs involved in the early stages of type 2 diabetes, such as miR-184 and miR-124a, associated with β-cell proliferation and differentiation, may serve as possible biomarkers [38•]. miR-124a reduces the levels of Ras-related Protein Rab-27a (Rab27A), a GTPase facilitating vesicle transport to the cell membrane, the direct binding of miR-124a to the 3′-untranslated region (3'-UTR) of Rab27A results in a decrease in Rab27A expression. Overexpression of miR-124a leads to excessive insulin release at rest and diminishes glucose-induced insulin release [10••]. In male mice, miRNA19a-3p levels decreased in the blood nine months before the onset of insulin resistance [25]. It has been found that miR-27a induces insulin resistance by inhibiting PPAR-ɣ and miR-27a levels are increased in pre-diabetic obese individuals [26]. This data indicates that miRNAs can be used as early biomarkers for individuals at risk of developing metabolic diseases later in life.

In a randomized controlled trial, changes in circulating levels of miRNAs in relation to type 2 diabetes or prediabetes status were investigated. The study included 462 patients without type 2 diabetes at baseline, and over a 60-month follow-up period, 107 individuals developed type 2 diabetes, 30 developed prediabetes, 223 maintained prediabetes, and 78 remained disease-free. The findings revealed higher baseline levels of miR-150 and miR-30a-5p, and lower levels of miR-15a and miR-375 in individuals with type 2 diabetes compared to healthy individuals. These miRNAs could serve as potential biomarkers to assess the risk of developing type 2 diabetes, thereby enhancing prediction and prevention strategies among individuals at high risk for the disease [14].

## Circulating miRNAs as Potential Diagnostic and Prognostic Biomarkers for Type 2 Diabetes

The expressions of miRNAs are altered in type 2 diabetes. In a case-control study, expression of miR-122 increased, while miR-126-3p and miR-146a expression levels decreased in type 2 diabetes patients compared to healthy individuals. These miRNAs may play a crucial role in the pathophysiology of type 2 diabetes through inflammatory pathways [41].

miR-155 plays a crucial role in maintaining β-cell function, with its upregulation indicating a β-cell adaptation to overnutrition. Disruption of this mechanism may be a key event in the transition from prediabetes to type 2 diabetes. Preclinical studies suggest that there is decreased expression of miR-155 observed in patients with type 2 diabetes. miR-155 downregulates genes involved in negative modulation of insulin receptor signaling and genes related to gluconeogenesis, while upregulating genes involved in glucose uptake in adipose tissue and skeletal muscle. Therefore, the decreased expression of miR-155 in type 2 diabetes patients may be one of the fundamental factors underlying the development of insulin resistance [42].

In a prospective cohort study, the findings revealed miR-192 increases in the serum of patients with prediabetes but does not change in the serum of patients with type 2 diabetes [8]. However, other studies have reported that serum levels miR-192 decreased in patients with type 2 diabetes [40]. Further research has posed that serum miR-192 levels are higher in patients with type 2 diabetes than in the control group [8]. These discrepancies indicate the need for more research to clarify the function of miR-192 in individuals with type 2 diabetes.

In a meta-analysis, the findings suggested that the expression of 40 miRNA species in patients with type 2 diabetes is irregular. Some miRNAs which upregulated (miR-29a, miR-34a, miR-375, miR-103, miR-107, miR-132, miR-142-3p, and miR-144) are identified as potential circulating biomarkers for type 2 diabetes, while others which down-upregulated (miR-199a-3p and miR-223) are thought to be tissue biomarkers [34]. Furthermore, it is essential to consider that specific miRNA profiles may be influenced by ethnicity-associated factors [4••].

Additionally, Takada et al. illustrated that the paired miRNAs, including miR‐10a and miR‐200c, or miR‐126 and miR‐10a, were altered in patients with type 2 diabetes compared to healthy individuals. The serum level of miR‐126‐3p was upregulated, while miR‐10a was downregulated. Their results indicated that paired miRNAs could be more effective diagnostics in patients with type 2 diabetes [43•].

The expression of some specific miRNAs changes in type 2 diabetes complications. Increased levels of circulating miR-25-3p and miR-320b, along with decreased levels of miR-495-3p, were observed in individuals with diabetic retinopathy compared to those without diabetic retinopathy and healthy individuals [44]. Moreover, miR-494-3p and miR-574-5p may regulate C7 protein expression, a diagnostic marker in early diabetic nephropathy [45]. Indeed, miR-155 has been implicated in the pathogenesis of various diabetic complications, including nephropathy, neuropathy, cardiomyopathy, and retinopathy. Additionally, it plays a role in the early stages of diabetic nephropathy. These findings suggest that miR-155 may serve as a potential therapeutic target for preventing or managing diabetic complications [42].

## Exploring the Impact of Phytochemicals on Type 2 Diabetes: A Focus on MicroRNA Regulation

Phytochemicals are effective on many diseases by changing the expression of miRNA. β-Sitosterol has been shown to be effective on non‐small cell lung cancer cells by inhibiting miR-181a-3p expression [46•], agathisflavone, a flavonoid, has been shown to be effective in neurodegenerative diseases including Alzheimer's disease by down-regulating miR146a and miR-155 [47•]. Mastiha, a natural supplement with active phytochemical, have anti-inflammatory effect by miRNA-155 regulation [48]. miR-155 expression is indeed decreased in type 2 diabetes and associated with various diabetes complications [42], targeting its inhibition through dietary interventions could hold therapeutic potential. However, further research is needed to elucidate the precise mechanisms involved and to determine the efficacy and safety of such dietary interventions in modulating miR-155 expression and mitigating type 2 diabetes and its complications.

**Fig. 3:**
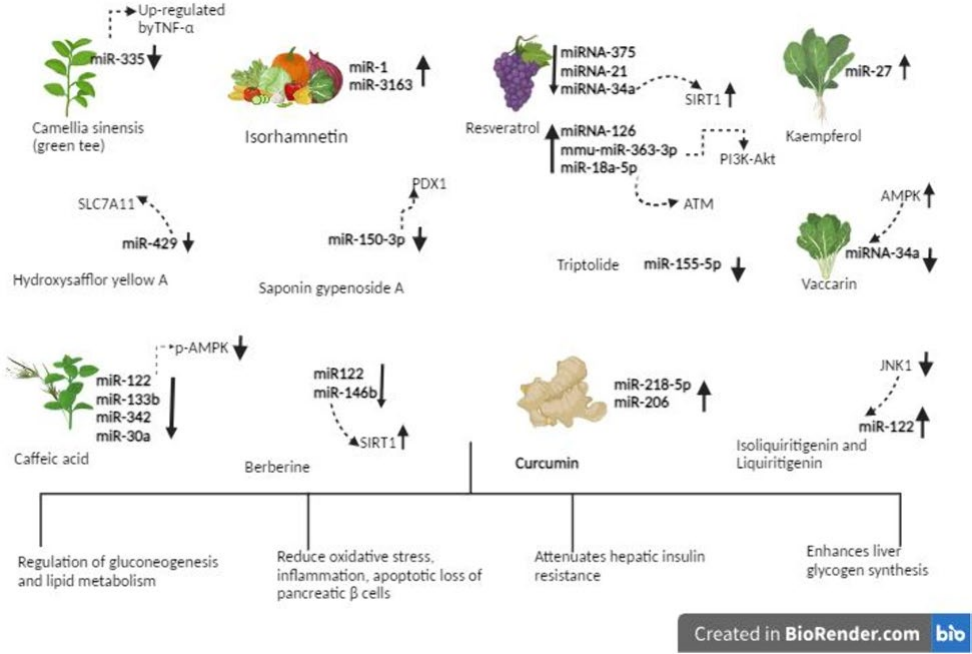
Phytochemicals are effective in type 2 diabetes by modulating the expression of miRNAs through pathways involved in insulin signaling and inflammation. TNF: Tumor Necrosis Factor, ATM: Ataxia-Telangiectasia Mutated Kinase, AMPK: AMP-Activated Protein Kinase, PDX1: Pancreatic and Duodenal Homeobox-1, JNK: c-Jun NH2-Terminal Kinase, SIRT1: Sirtuin 1, PI3K/AKT: Phosphatidylinositol 3-Kinase (PI3K)/Protein Kinase B, SLC7A11: Solute Carrier Family 7a Member 11

miRNAs can be proposed as biomarkers of type 2 diabetes, which could be regulated in response to strategies and treatments for type 2 diabetes pathologies. Dietary components such as phytochemicals may be fundamental treatments, particularly in type 2 diabetes. Studies have suggested that phytochemicals may be effective in type 2 diabetes by influencing ncRNA expression [4••, 10••] (Fig. 3).

Green tea is a plant that contains high concentrations of flavonoids, such as catechins and other polyphenolic compounds. When mice fed a high-fat diet were administered 500 mg/body weight of green tea polyphenols for 12 weeks, a decrease in miR-335 expression in adipose tissue was observed. miR-335, which TNF-α up-regulates in adipocytes, may serve as a link between inflammation and impaired metabolism in adipose tissue [31]. In a randomized, double-blind, crossover study, obese women were instructed to take two capsules of green tea (738 mg). Following consumption, circulating miRNA expression was evaluated. It was observed that a high-fat, high-saturated meal increased the expression of 62 miRNAs 5 h after consumption. However, green tea inhibited the expression of miRNAs induced by the high-fat, high-saturated meal intake [49••].

Isorhamnetin, a quercetin metabolite, and its O-methylated flavonol abundant in blackberries, pears, apples, cherries and in medicinal herbs, has many pharmacological actions involving anti-inflammatory, antioxidant, anticancer, anti-obesity, antidiabetic, antimicrobial hepatoprotective, and neuroprotective effects [50, 51]. Isorhamnetin also have epigenetic regulators, it up-regulates the expression of AKT2 mRNA, which regulates glucose uptake, fatty acid transport, glycogen synthase activity, and insulin resistance. Additionally, isorhamnetin increased the expression of miR-1 and miR-3163 [50]. Isorhamnetin regulates autophagy-related genes and their epigenetic regulators. In a rat model of diabetic nephropathy, it significantly decreased the expression of miR-34a, miR-15b, and miR-633 in kidney tissues. These findings suggest that isorhamnetin exhibits renoprotective and therapeutic effects in diabetic nephropathy [51]. These findings suggest that isorhamnetin could serve as an alternative or potential complementary treatment for type 2 diabetes and its complications [50, 51]. Furthermore, the combination of epigallocatechin gallate and quercetin protects islet cells by reducing the expression of miR-16-5p in pancreatic β cells [52]. This combination modulates the regulation of miR-27a-3p and miR-96–5p, directly affecting FOXO1 expression and improving hepatic gluconeogenesis and insulin resistance [53••].

Resveratrol, a natural polyphenol found in various plant sources like berries, grapes, and peanuts, has been shown to have diverse effects on cellular processes and gene expression, including the modulation of miRNAs. These miRNAs play crucial roles in inflammation, cell proliferation, homeostasis, and metabolic diseases [54, 55]. Resveratrol's ability to influence miRNA expression suggests its potential in regulating various cellular pathways involved in health and disease. By targeting specific miRNAs, resveratrol may exert anti-inflammatory, anti-proliferative, and metabolic effects, offering potential therapeutic benefits for conditions such as metabolic diseases, including obesity and type 2 diabetes [55, 56••, 57–61]. In a randomized, double-blind, placebo-controlled study, resveratrol supplementation for six months increased miRNA-126 expression and decreased miRNA-375, miRNA-21, and miRNA-34a expressions in individuals with type 2 diabetes [56••]. Resveratrol has been reported to reduce oxidative stress and inflammation by increasing miRNA-126 expression [57]. Decreased expression of miRNA-375 has demonstrated effectiveness in managing type 2 diabetes by lowering the apoptotic loss of pancreatic β cells and maintaining adequate insulin secretion in the body [58]. Additionally, miRNA-21 has been found to mitigate oxidative stress by reducing hydrogen peroxide-induced reactive oxygen species production and glycolysis process in pancreatic cells through resveratrol application [55]. Moreover, decreased expression of miRNA-34a with resveratrol administration increased the expression of Sirtuin 1 (SIRT1), which has a protective role in reducing oxidative stress and inflammation in type 2 diabetes [59]. In mice with induced insulin resistance due to a high-fat diet, resveratrol mitigated insulin resistance by inducing the overexpression of mmu-miR-363-3p in the phosphatidylinositol 3-kinase (PI3K)/protein kinase B (PI3K/AKT) signaling pathway [60]. miRNAs play a crucial role in the regulation of the autophagy pathway. The administration of resveratrol to db/db mice at 100 mg/kg once daily for 12 weeks resulted in a significant upregulation of miR-18a-5p. This resveratrol-induced increase in miR-18a-5p expression effectively prevents and alleviates chronic diabetic nephropathy by regulating autophagy activity through Ataxia-telangiectasia mutated kinase (ATM) [61]. However, while promising, further research is needed to fully understand the mechanisms by which resveratrol affects miRNA expression and how these changes translate into physiological outcomes. Additionally, the bioavailability and optimal dosage of resveratrol for achieving therapeutic effects need to be carefully considered in future studies and potential clinical applications.

Caffeic acid, a phenolic compound of hydroxyl cinnamic acid, found in many vegetables such as berries, kiwis, apples plums and also coffee. Caffeic acid have cardiovascular protective effects, hypoglycemic, antibacterial, antioxidant, anti-inflammatory, and anticancer effects [62••, 63]. The administration of caffeic acid (50 mg/kg/day) to diabetic hyperlipidemic rats significantly inhibited the expression of miR-122 and phosphorylated AMP-activated protein kinase (pAMPK) activation. This inhibition could block the activity of essential enzymes involved in synthesizing cholesterol and fatty acids [62••]. Caffeic acid modulates the autophagy pathway by inhibiting autophagy regulatory miRNAs (miRNA-133b, -342, and 30a), and this modulation has been reported to have ameliorative effects against diabetic kidney disease [63].

Ellagic acid, a natural polyphenolic compound found in various plant extracts, fruits, and nut exhibits numerous biological properties, including antidiabetic, anti-inflammatory, antioxidant, and hepatoprotective activities. Studies have shown that ellagic acid can activate miR-223, which may serve as a marker of oxidative stress. Furthermore, it targets kelch-like ECH-associated protein 1 (keap1), a crucial regulator of the antioxidant response and glucose metabolism, inhibiting its expression in HepG2 cells exposed to high glucose levels [64].

Berberine, a quaternary ammonium salt from the protoberberine group of isoquinoline alkaloids, reduces weight gain, enhances glucose metabolism by inhibiting gluconeogenesis, and have anti-hyperglycaemic, anti-hyperlipidaemic effects [65]. Berberine down-regulates Beclin 1 (BECN1), a protein associated with autophagy through the miR30 family, in adipocytes of mice fed a high-fat diet. The regulation of BECN1 may represent a crucial mechanism in controlling autophagy in adipocytes during obesity, a significant contributor to the development of type 2 diabetes [66]. Berberine administration to diabetic male C57BL/6 J mice at both low-dose (40 mg/kg) and high-dose (160 mg/kg) resulted in dose-dependent decreases in miR122 expression in the liver of mice, with berberine regulating gluconeogenesis and lipid metabolism [67]. Berberine attenuates hepatic insulin resistance and enhances liver glycogen synthesis by downregulating miR-146b and increasing SIRT1 expression. This effect occurs through the deacetylation of FOXO1 in high-fat diet-fed mice (5 mg/kg/day) and palmitic acid-treated HepG2 cells (10 µM for 24 h) [22].

Curcumin which is extracted from turmeric known for its potent anti-inflammatory, antioxidant, anticancer neuroprotective properties and protective role in several diseases including nervous system disorders, cancer, cardiovascular disease, and other common disease [68••, 69]. Additionally, research has shown that curcumin can decrease the inflammatory response by increasing miR-218-5p expression in pheochromocytoma cells, opening it up to potential usage in the future treatment of diabetic encephalopathy [68••]. In rats with fructose-induced insulin signaling disorders, 60 mg/kg curcumin administration was reported to alleviate insulin signaling dysfunction by increasing miR-206 expression [69].

Triptolide, a diterpenoid, prevents oxidative stress and inflammatory damage by upregulating brain-derived neurotrophic factor (BDNF) through downregulating miR-155-5p in a mouse model of diabetic nephropathy [70]. In addition, triptolide exhibits efficacy in diabetic renal diseases through the regulation of miR-137 [71], miR-141-3p [72], and miR-188-5p expression [73]. In a cell study, it has been reported that the natural triterpenoid compound celastrol reduces insulin resistance induced by palmitic acid through the modulation of miR-223 [74].

Furthermore, phytochemicals such as Hydroxysafflor yellow A, liquiritigenin, kaempferol saponin genocide A, saccharin, and quercetin have been shown to modulate miRNAs in the context of type 2 diabetes [75••, 76, 77, 78••, 79]. In a mouse model of diabetic atherosclerosis, the administration of Hydroxysafflor yellow A, a single chalcone glycoside, at a dosage of 2.25 mg/kg for 12 weeks, led to the downregulation of miR-429 expression, which in turn regulates solute carrier family 7a member 11 (SLC7A11) expression [75••]. It was observed that miR-122 expression decreased in the liver of mice fed a high-fat diet for 11 weeks and in HepG2 cells treated with 20 ng/mL TNF-α. Additionally, isoliquiritigenin and liquiritigenin flavonoids found in liquorice have been shown to increase miR-122 expression by inhibiting JNK1 activation [76]. In high-fat diet-induced obesity mice, kaempferol, a semi-synthesized flavonoid derivative of tiliroside, increased the expressions of pAMPK and miR-27 in the liver and fatty tissues [77]. Administration of saponin gypenoside A at doses of 50 or 100 mg/kg/d for 12 weeks to high-fat diet-fed mice inhibited miR-150-3p expression. This miRNA is closely related to the dysfunction of pancreatic β cells by targeting PDX1. Consequently, the restored level of PDX1 was observed [78••]. Vaccarin, an active flavonoid glycoside, inhibits the upregulation of miRNA-34a triggered by high glucose levels. It was shown that vaccarin activates the expression of AMPK, which downregulates miRNA-34a [79].

Betaine, also known as N-trimethylglycine, is a naturally occurring substance abundant in whole grains, spinach, and shellfish. Betaine can modulate gut microbiota composition and promote the production of short-chain fatty acids (SCFAs). Additionally, it has been proposed that betaine may influence the miR-378a family by regulating the DNA methylation of its promoter region [80].

These studies suggest that polyphenols may exert control over type 2 diabetes by regulating various miRNAs, indicating a potential therapeutic strategy for enhancing the management of type 2 diabetes. Further studies are warranted to substantiate and verify the mechanisms underlying the action of phytochemicals in mitigating or averting diabetes and its complications by modulating miRNA expressions in individuals diagnosed with type 2 diabetes.

## Conclusion

MiRNAs play vital roles in developmental processes, cell defense and differentiation, DNA replication, transcription, and post-transcriptional processes. Numerous studies have highlighted the potential of ncRNAs as therapeutic targets, given their involvement in regulating various cellular functions. Recent studies have shown that miRNAs play essential roles in insulin synthesis and secretion, the regulation of β-cell function, and glucose metabolism, thus contributing to the development of insulin resistance and type 2 diabetes. These findings indicate that miRNAs hold promise as novel therapeutic biomarkers for diagnosing and treating type 2 diabetes. While association studies have predominantly supported using miRNAs as biomarkers for type 2 diabetes, it is worth emphasizing that correlation does not imply causality, necessitating large-scale clinical studies for validation.

Manipulating the expression of miRNAs offers a new avenue for investigating different diagnoses and treatments for numerous diseases. Identifying novel miRNAs, particularly those involved in energy metabolism and insulin secretion, will shed light on the pathogenesis of type 2 diabetes. Therefore, many miRNA functions remain undiscovered, potentially offering new perspectives in diagnosing and treating type 2 diabetes within clinical, genetic, and epigenetic fields. The effects of phytochemicals on type 2 diabetes and its complications through miRNAs are multifactorial. Further research is warranted to elucidate the mechanisms underlying the action of phytochemicals in reducing or preventing diabetes complications by regulating miRNA expression in individuals with type 2 diabetes. Additionally, comprehensive studies are necessary to assess the safety and efficacy of phytochemicals and their combination in clinical trials. Considering the capacity of phytochemicals to modulate ncRNA expression, it may be beneficial to incorporate foods rich in phytochemicals and eat a rainbow of plant-based foods into the diet.

## Data Availability

No datasets were generated or analysed during the current study.
